# Sodium butyrate promotes the function of *NDUFS2* in bovine skeletal muscle fiber type transformation and mitochondrial biosynthesis

**DOI:** 10.3389/fvets.2026.1747515

**Published:** 2026-03-17

**Authors:** Xiaonan Zhou, Junjie Xu, Chang Qu, Zhiyan Zhao, Yanling Ding, Chenglong Li, Zonghua Su, Xiaolong Kang

**Affiliations:** 1 College of Animal Science and Technology, Ningxia University, Yinchuan, China; 2 Yinchuan Animal Husbandry Technical Extension and Service Center, Yinchuan, China

**Keywords:** bovine skeletal muscle cells, mitochondria, muscle fiber type, *NDUFS2*, sodium butyrate

## Abstract

**Methods:**

In this study, the function of *NDUFS2* in BSMCs was investigated through gene manipulation. Additionally, cells were treated with sodium butyrate (NaB) to assess its regulatory effect on *NDUFS2*. Key parameters, including cell proliferation and differentiation, myofiber type conversion, ATP levels, and mitochondrial DNA (mtDNA) copy number, were measured. Protein-protein interactions were also analyzed.

**Results:**

*NDUFS2* promoted BSMC proliferation while inhibiting differentiation. It facilitated the conversion of fast-twitch to slow-twitch muscle fibers and enhanced cellular energy metabolism, as evidenced by increased ATP and mtDNA copy number. NaB treatment further augmented the functionality of *NDUFS2* and validated its interaction with the NDUFS1 protein.

**Discussion:**

This study demonstrates that NaB treatment regulates *NDUFS2* function, impacting mitochondrial biosynthesis and myofiber type transformation. These findings provide a theoretical basis for the molecular regulation of muscle phenotype in domestic animals.

## 1 Introduction

Skeletal muscle is derived from the differentiation of satellite cells into myoblasts, a process regulated by multiple cytokines and signaling pathways (1–3). It is also a tissue rich in mitochondria, organelles essential for energy metabolism and ATP synthesis (4, 5). Mitochondrial content directly influences the oxidative capacity of muscle and is a key determinant of myofiber type (6). For example, interventions that promote the switch of muscle fibers to an oxidative phenotype (e.g., baicalin treatment) are usually accompanied by upregulation of mitochondrial complex expression and activity (7). Type I (slow-twitch) fibers contain abundant mitochondria, appear reddish due to high myoglobin content, primarily rely on fat oxidation for ATP production, and exhibit high oxidative capacity (8). In contrast, type II (fast-twitch) fibers possess fewer and less active mitochondria, appear lighter in color, and pre-dominantly depend on glycolysis for rapid ATP generation (9). Butyrate is produced by microbial fermentation of fibers in the animal gut (10, 11) and is a known histone deacetylase inhibitor (HDACi) (12). It promotes the expression of mitochondrial biogenesis-related genes (such as *PGC-1*α) through epigenetic modifications (13). NaB participates in multiple metabolic pathways, regulating skeletal muscle development and mitochondrial biosynthesis (14, 15). Adding butyrate to the diet upregulates the expression of myosin heavy chain I (*MYHC I*) and *MYHC II a* genes in the gastrocnemius muscle of mice, increases the levels of slow-twitch fiber markers, myoglobin, and troponin, and promotes the formation of oxidative muscle fibers (16). Therefore, investigating how sodium butyrate modulates the mitochondrial function and fiber-type composition in skeletal muscle provides a critical perspective for understanding the crosstalk between cellular metabolism and muscle development.

The mitochondria-associated gene *NDUFS2* (NADH dehydrogenase ubiquinone iron-sulfur protein 2) is located in the inner membrane of mitochondria. *NDUFS2* is crucial for cell growth and development, intercellular signaling, and participation in the metabolic processes of the organism through mitochondrial biosynthesis (17). Additionally, *NDUFS2* has been implicated in organismal oxygen sensing (18). Inhibition of *NDUFS2* in pancreatic cancer cell lines leads to mitochondrial damage and functional impairment (19). In pulmonary artery smooth muscle, *NDUFS2* contributes to the generation of ROS (20). Mutations in *NDUFS2* significantly affect neural stem cell proliferation and ATP synthesis, shifting cellular metabolism toward glycolysis (21). Overall, *NDUFS2* is involved in the regulation of cell proliferation and viability, complex I-related oxidative phosphorylation efficiency, glycolytic metabolism, intracellular ATP reserves, and cell membrane structural integrity (22). However, how *NDUFS2* works in BSMCs remains unknown.

Based on the above research findings, we hypothesize that an as-yet-unidentified interaction exists between the endogenous mitochondrial function-related gene (*NDUFS2*) and the exogenous metabolic modulator (NaB). This study demonstrates through a series of experiments that NaB and *NDUFS2* synergistically co-regulate mitochondrial function in bovine skeletal muscle cells, thereby influencing their myofiber type conversion. We anticipate that these findings will provide a theoretical basis for the application of exogenous small molecules within agriculture.

## 2 Materials and methods

### 2.1 Isolation and culture of bovine muscle cells

The longissimus dorsi muscle of calves was collected and disinfected through a rapid rinse with alcohol, followed by a brief wash in PBS (containing 2% penicillin-streptomycin). Subsequently, the BSMCs were isolated using the type I collagenase method and further purified through differential centrifugation, as previously study (14). This animal experiment received approval from the Science and Technology Ethics Committee of Ningxia University (24-F-062).

### 2.2 siRNA synthesis, overexpression vector construction, and transfection

To investigate the function of the *NDUFS2* in BSMCs, four siRNA sequences of the *NDUFS2* were designed and synthesized in the course of this experiment. The synthesis of the siRNA fragments was conducted by Shanghai Sangon Biotech, and the relevant sequence information is provided in Supplementary Table S1. The most efficacious interference was observed with the btaNDUFS2-401, which was named si-NDUFS2 primers for *NDUFS2* gene were developed using the Primer Premier 5 software (version: 6.25), and the pcDNA3.1 vector of *NDUFS2* gene was synthesized by Shanghai Sangon Biotech.

The cells were inoculated in six-well cell culture plates at a concentration of 11 × 0^5^ cells/ml. After the cells adhered to the surface and reached a confluence of approximately 70–80%, the original culture medium was discarded 24 h before the transfection procedure. This medium was replaced with Opti-MEM (Gibco, USA), which is devoid of antibiotics but contains an appropriate amount of serum. The plasmids pcDNA3.1 (NC-EX) and pcDNA3.1-NDUFS2 (EX-NDUFS2) were then mixed with the transfection reagents according to the instructions provided with the Lipofectamine™ 3000 Transfection Reagent (catalog no. L3000008, ThermoFisher, USA). The resulting complexes were incubated at room temperature for 20 min before being added dropwise to the cell culture dishes. Following a 48 h incubation period, the relevant biological indicators were analyzed.

### 2.3 EdU assay

Cell proliferation was detected with BeyoClick™ EdU Cell Proliferation Kit with Alexa Fluor 594 (catalog no. C0078S, Beyotime, China). When the cell density to 50–70% for transfection, transfection to the cell 48 h, configure, add 1 × of EdU working solution, incubated for 2 h, using fixation solution at room temperature fixed for 15 min, after that add the appropriate amount of permeabilization solution, the room continues to incubate for 10–15 min, cell washing and add Click reaction solution, placed at room temperature and incubated for 30 min away from the light. The cell nuclei were stained with Hoechst 33342 solution (catalog no. C1025, Beyotime) and incubated at room temperature for 10 min, wash three times with the washing solution for 3–5 min each, and then analyzed by eclipse E600 fluorescence microscope (Nikon, Japan). Five views were randomly selected from each well and the ratio of the number of positive cells to the total number of BSMCs cells was subsequently calculated with ImageJ software (version: 1.46r).

### 2.4 Mitochondrial metabolism-related indicator assay

ATP levels were measured using the ATP Assay Kit (catalog no. S0026, Beyotime). The procedure is outlined as follows: thaw and homogenize the frozen fluorescent assay, ensuring that its temperature reaches room temperature. Remove the cell culture plate and allow it to equilibrate at room temperature for 10 min. The 96-well plates were then lysed by adding 100 μl of assay reagent to each well, followed by shaking at room temperature for 2 min. The cells were incubated at room temperature for 10 min, and chemiluminescence was measured at 450 nm using a multifunctional enzyme reader. Based on the chemiluminescence data, the relative activity of the cells was calculated.

ROS level analysis was performed when the cell confluence reached approximately 70–80%. Before the probe loading operation, the DCFH-DA solution (catalog no. S0033S-1, Beyotime) was diluted 1:1,000 in serum-free culture medium to a final concentration of 10 μm. The original culture medium was then aspirated and replaced with DCFH-DA solution, ensuring the volume was sufficient to completely cover the cell monolayer. Cells were subsequently incubated in the dark at 37 °C for 20–30 min. Fluorescence intensity changes were measured using an Eclipse E600 fluorescence microscope (Nikon) with excitation at 488 nm and emission at 525 nm.

Mitochondrial membrane potential was assessed with Mitochondrial Membrane Potential Kit (catalog no. C1071S, Beyotime). Briefly, the Rhodamine 123 staining working solution was prepared by diluting 1 μl of the high-concentration stock solution (1,000 × ; catalog no. C2007, Beyotime) in 1 ml of assay buffer. The culture medium in the 96-well plate was aspirated, and the wells were washed with PBS. Subsequently, 100 μl of the Rhodamine 123 working solution was added to each well, and the plate was incubated in the dark at 37 °C for 20–60 min. After incubation, the supernatant was removed, and the cells were washed twice with pre-warmed culture medium. Fluorescence intensity was measured using an Eclipse E600 fluorescence microscope (Nikon) with excitation at 507 nm and emission at 529 nm.

The mtDNA copy number was determined as follows: after transfection, total cellular DNA was extracted, and a standard curve was established. Absolute quantification was then performed by measuring the Ct value of *NDUFS2*, and the mtDNA copy number was calculated by substituting the Ct value into the standard curve.

### 2.5 RNA extraction, reverse cDNA, and qPCR

Cellular RNA was extracted using RNAiso Plus (catalog no. 9109, Takara, Japan), and the integrity of total RNA was assessed through 1% agarose gel electrophoresis. The concentration of total RNA and the OD_260/280_ values were measured with a spectrophotometer (ThermoFisher, USA). Total RNA was reverse transcribed into cDNA with PrimeScript™ RT Reagent Kit (Perfect Real Time; catalog no. RR037A, Takara) (23), with the following reaction program: 37 °C, 15 min, 85 °C, 5 s. The amplified and synthesized cDNA stock solution was diluted and stored at −20 °C for future use. RT-qPCR was conducted using TB Green^®^ Premix Ex Taq™ II FAST qPCR (catalog no. CN830A, Takara), with primer information provided in Supplementary Table S2. Relative expression levels were determined via the 2^−Δ*ΔCt*^ method (24), with the β*-actin* gene serving as the endogenous control. Each experiment included a minimum of three technical replicates.

### 2.6 Western blot assay

Whole cell proteins were extracted using the Whole Protein Extraction kit (catalog no. KGB5303-50, KeyGEN, China). The protein concentration was determined using the BCA method. Following denaturation, electrophoresis, and membrane transfer, the samples underwent incubation with primary and secondary antibody, respectively. The cells were processed using the Affinity™ ECL Kit (femtogram; catalog no. KF8003, Affinity Biosciences, USA). Finally, protein bands were visualized using a Tanon 5200 Multi fully automated chemiluminescence image analysis system (Tanon, Shanghai, China) and analyzed for grayscale intensity and relative expression using ImageJ software (version: 1.46r), as detailed in a previous study (14). The antibody information is provided in Supplementary Table S3.

### 2.7 RNA-seq analysis

Total RNA was extracted from BSMCs (*n* = 4) in both the control and si-NDUFS2 groups using the TRIzol reagent (Invitrogen). After verifying of RNA quality, sequencing libraries were constructed. Library construction was initially quantified using a Qubit 3.0 Fluorometer (Thermo Fisher Scientific), with a minimum requirement of 1 ng/μl. The insert fragments in the library were assessed using a Qsep400 high-throughput analysis system. Upon confirmation of expected insert sizes, quantitative PCR (qPCR) was performed to determine the effective library concentration, ensuring it exceeded 2 nm for quality control. High-throughput sequencing was carried out in PE150 mode on a sequencing platform. Following sequencing, raw data were filtered to yield clean data, which were then aligned to the bovine reference genome (ARS_UCD2.0). Differential expression analysis was conducted with a threshold of fold change ≥2 and an FDR < 0.01. GSEA (Gene Set Enrichment Analysis), GO (Gene Ontology) annotation, and KEGG (Kyoto Encyclopedia of Genes and Genomes) enrichment analyses were performed on the DEGs (2). For validation, 10 randomly selected DEGs were subjected to qPCR analysis (e with at least three technical replicates per sample), and their relative expression levels calculated using the 2^−Δ*ΔCt*^ method (25).

### 2.8 RNA-pull down

The full-length coding region of *NDUFS2* was cloned into the pUC57 vector using the MAXIscript^®^ kit (Thermo Fisher Scientific) and following the manufacturer's instructions. The Transcription Aid T7 High Yield Transcription Kit (Thermo Fisher Scientific) was employed to transcribe full-length sense and antisense RNA sequences of *NDUFS2 in vitro*. These sequences were subsequently purified using the GeneJET RNA Purification Kit (Thermo Fisher Scientific). The purified sense and antisense RNA sequences of *NDUFS2* were labeled with 3'-desulfurized biotin at the 3'-end using the Pierce RNA 3'-End Desulfurized Biotin Labeling Kit (Thermo Fisher Scientific). Subsequently, the complex was purified using the Pierce Magnetic Pull-Down Kit (Thermo Fisher Scientific) and then separated by SDS-PAGE. The isolated proteins were stained using the Rapid Silver Stain Kit (Beyotime), followed by mass spectrometry to identify specific proteins interacting with *NDUFS2*.

### 2.9 Statistics and analysis

Data were analyzed and visualized using GraphPad Prism 8.0.2. *P* < 0.05 indicates a significant difference, which is indicated by “^**^”, *P* < 0.01 indicates an extremely significant difference, which is indicated by “^***^”, *P* > 0.05 indicates no significant difference, which is indicated by “ns”.

## 3 Results

### 3.1 *NDUFS2* promotes proliferation and inhibits differentiation of BSMCs

**Figure 1:**
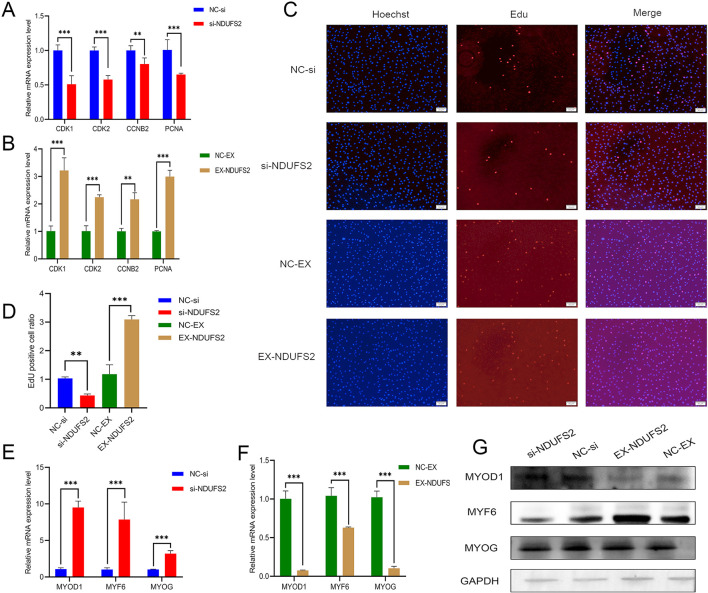
Effect of NDUFS2 gene on the proliferation and differentiation of BSMCs. **(A)** Effect of *NDUFS2* gene interference on proliferation marker genes. **(B)** Effect of *NDUFS2* gene overexpression on proliferation marker genes. **(C)** EdU staining of BSMCs, EdU (red) staining, and nuclear (blue) staining (scale = 200 μm). **(D)** Statistics of positive cell ratios. **(E)** Effect of *NDUFS2* gene interference on differentiation marker genes. **(F)** Effect of *NDUFS2* gene overexpression on differentiation marker genes. **(G)** Western blot analysis of the effect of *NDUFS2* gene on the differentiation of BSMCs. Significance levels are indicated as follows: “ns” for *P* > 0.05, “**” for *P* < 0.05, “***” for *P* < 0.01.

To investigate the function of *NDUFS2*, an siRNA sequence targeting *NDUFS2* and an overexpression vector of *NDUFS2* gene were designed and assessed for efficiency (Supplementary Figure S1). Following *NDUFS2* interference, a significant downregulation of *CDK1, CDK2, CCNB2*, and *PCNA* showed significant downregulation at the mRNA level (*P* < 0.01; Figure 1A). The overexpression of *NDUFS2* promoted the mRNA level of these genes (*P* < 0.01; Figure 1B). Furthermore, the EdU assay revealed that *NDUFS2* overexpression significantly promoted cellular proliferation (*P* < 0.01; Figures 1C, D). Collectively, these findings suggest that *NDUFS2* plays a crucial role in promoting the proliferation of BSMCs. The mRNA level of differentiation marker genes *MYOD1, MYF6*, and *MYOG* were significantly increased following *NDUFS2* gene interference, compared to the control group (*P* < 0.001; Figure 1E). In contrast, overexpression of *NDUFS2* resulted in a marked suppression of its mRNA levels (Figure 1F). Western blot analysis revealed that *NDUFS2* inhibited the protein levels of MYOD1, MYF6, and MYOG (Figure 1G). These results indicate that *NDUFS2* may contribute to the inhibition of BSMC differentiation.

### 3.2 *NDUFS2* regulates muscle fiber types in BSMCs

**Figure 2:**
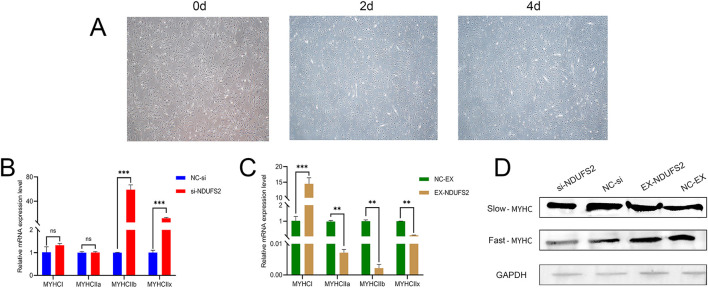
Effect of *NDUFS2* gene on myofiber type. **(A)** When cell density reached 90%, differentiation medium was employed to induce differentiation of bovine skeletal muscle satellite cells. After 2 days, a small number of myotubes had formed. By day 4, BSMCs had fused to form numerous myotubes (scale = 500 μm). **(B)** Effect of interfering with *NDUFS2* gene on the myofiber type marker gene. **(C)** Effect of overexpressing *NDUFS2* gene on the myofiber type marker gene. **(D)** Western blot analysis of *NDUFS2* gene in myofiber type in BSMCs. Significance levels are indicated as follows: “ns” for *P* > 0.05, “**” for *P* < 0.05, “***” for *P* < 0.01.

Differentiation of BSMSCs for 4 days using 2% horse serum was conducted to investigate myofiber type transition (Figure 2A). Interference with NDUFS2 significantly increased the mRNA levels of *MYHC IIb* and *MYHC IIx* genes (*P* < 0.001), while the mRNA levels of *MYHC I* and *MYHC IIa* remained unaffected (*P* > 0.05; Figure 2B); Conversely, overexpression of *NDUFS2* significantly elevated the mRNA levels of *MYHCI* (*P* < 0.001), and markedly decreased the mRNA levels of *MYHC IIa, MYHC IIb*, and *MYHC IIx* genes (*P* < 0.01; Figure 2C). Western blot analysis revealed that interference with *NDUFS2* gene inhibited the protein levels of slow-MYHC while promoting fast-MYHC, consistent with the findings from *NDUFS2* overexpression (Figure 2D). qPCR analysis demonstrated that the mRNA levels of the *NDUFS2* gene were highest in the longissimus dorsi muscle of cattle (*P* < 0.05), followed by the biceps femoris muscle (*P* < 0.01; Supplementary Figure S2A). This indicates tissue-specific mRNA levels of *NDUFS2* gene across major tissues in bovine organisms. Compared to calves, adult cattle exhibited significantly higher mRNA levels of *NDUFS2* in the longissimus dorsi and biceps femoris muscles (*P* < 0.05; Supplementary Figure S2B). This suggests temporal specificity in the gene's expression during bovine muscle development, potentially conferring a crucial biological role in governing myofiber formation in mature cattle.

### 3.3 *NDUFS2* regulates the function of BSMCs mitochondria

**Figure 3:**
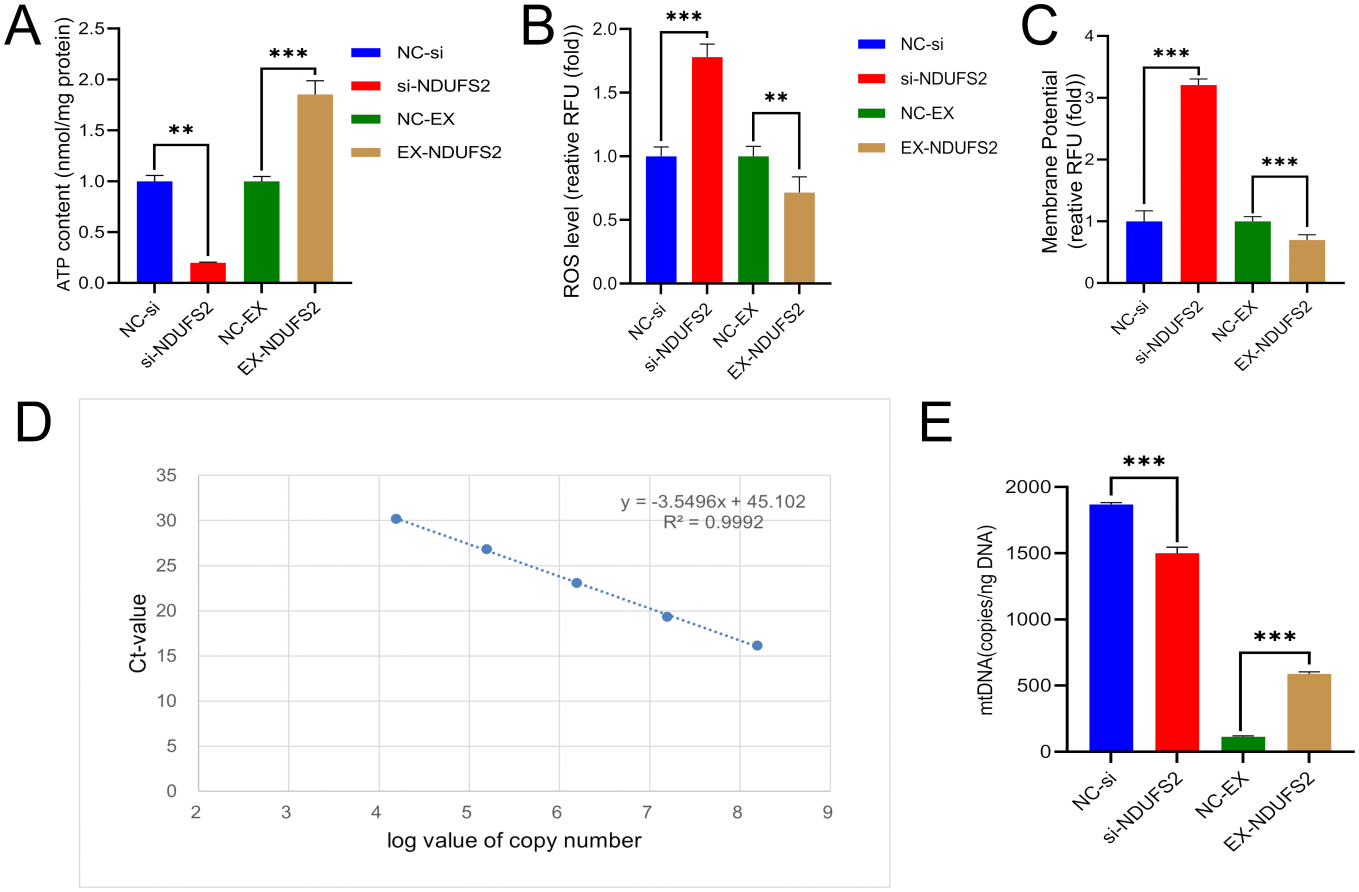
Effect of *NDUFS2* gene on mitochondrial function in BSMCs. **(A)** ATP level. **(B)** ROS level. **(C)** Membrane potential. **(D)** *NDUFS2* gene standard curve. **(E)** Mitochondrial mtDNA copy number. Significance levels are indicated as follows: “ns” for *P* > 0.05, “^**^” for *P* < 0.05, “^***^” for *P* < 0.01.

ATP levels significantly decreased (*P* < 0.01) following *NDUFS2* interference, while they markedly increased (*P* < 0.001) with *NDUFS2* overexpression (Figure 3A). ROS levels were extremely significantly elevated (*P* < 0.001) with *NDUFS2* interference and significantly reduced (*P* < 0.01) with *NDUFS2* overexpression (Figure 3B). Membrane potential was extremely significantly increased (*P* < 0.001) with *NDUFS2* interference and significantly decreased (*P* < 0.001) with *NDUFS2* overexpression (Figure 3C). Additionally, the mtDNA copy number was extremely significantly decreased (*P* < 0.001) with *NDUFS2* interference and extremely significantly increased (*P* < 0.001) with *NDUFS2* overexpression (Figures 3D, E). These aforementioned results indicate that *NDUFS2* gene can enhance mitochondrial ATP levels and mtDNA copy numbers, while inhibit mitochondrial ROS generation and inhibit the mitochondrial membrane potential (MMP).

### 3.4 NaB potentiates the inhibitory effect of *NDUFS2* on BSMC differentiation

**Figure 4:**
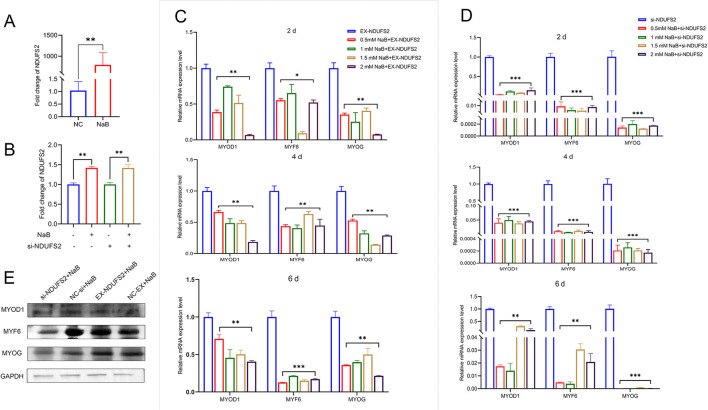
NaB and *NDUFS2* genes jointly regulate BSMCs differentiation. **(A)** NaB promotes *NDUFS2* gene mRNA level. **(B)** NaB can reverse the effects of *NDUFS2* gene interference. **(C)** NaB and EX-NDUFS2 co-treatment in the differentiation of BSMCs. **(D)** NaB and si-NDUFS2 co-treatment in the differentiation of BSMCs. **(E)** Western blot analysis to detect the effect of *NDUFS2* gene and NaB co-treatment on the differentiation of BSMCs. Significance levels are indicated as follows: “ns” for *P* > 0.05, “^**^” for *P* < 0.05, “^***^” for *P* < 0.01.

NaB promotes the expression of *NDUFS2* gene (*P* < 0.01; Figure 4A). In the presence of *NDUFS2* interference, the addition of NaB effectively reverses these changes (*P* < 0.01; Figure 4B). To assess the impact of NaB on the differentiation of BSMCs following co-treatment with *NDUFS2* gene, a range of NaB concentrations (0 mm, 0.5 mm, 1 mm, 1.5 mm, and 2 mm) was applied, with each concentration administered at 0, 2, 4, and 6 days. qPCR results indicated that co-treatment with *NDUFS2* overexpression and NaB significantly reduced in the mRNA levels of *MYOD1, MYF6*, and *MYOG* (*P* < 0.05; Figure 4C). Furthermore, the interference of NDUFS2 in conjunction with NaB similarly resulted in decreased differentiation (*P* < 0.05; Figure 4D). Western blot analysis revealed that *NDUFS2* increased the protein level of MYOD1, MYF6, and MYOG (Figure 4E).

### 3.5 NaB exacerbates *NDUFS2* regulation of muscle fiber type in BSMCs

**Figure 5:**
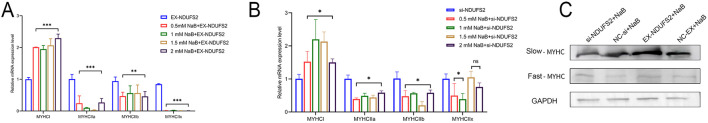
Effect of NaB on *NDUFS2* gene co-treatment in the transformation of fiber types in BSMCs. **(A)** NaB and EX-NDUFS2 co-treatment. **(B)** NaB and si-NDUFS2 co-treatment. **(C)** Western blot to detect the effect of *NDUFS2* gene and NaB co-treatment on the myofiber types of BSMCs. Significance levels are indicated as follows: “ns” for *P* > 0.05, “^**^” for *P* < 0.05, “^***^” for *P* < 0.01.

BSMCs were co-treatment with different concentrations of NaB and *NDUFS2* to induce to differentiation. The qPCR results indicated that overexpression of *NDUFS2* significantly increased the mRNA level of *MYHCI* (*P* < 0.05), while decreasing the mRNA levels of *MYHC IIa, MYHC IIb*, and *MYHC IIx* (*P* < 0.01; Figure 5A). Conversely, interference with *NDUFS2* led to a significant increase in the mRNA level of *MYHCI* (*P* < 0.05), and a significant decrease in the mRNA levels of *MYHC IIa, MYHC IIb, and MYHC IIx* (*P* < 0.05; Figure 5B). Western blot analysis revealed that *NDUFS2* reduced the protein level of fast-MYHC and increased the level of slow-MYHC (Figure 5C). These results suggest that the synergistic regulation of *NDUFS2* and NaB facilitates the transition of myofibrillar types in BSMCs.

### 3.6 NaB exacerbates the mitochondrial regulation of BSMCs by the *NDUFS2*

**Figure 6:**
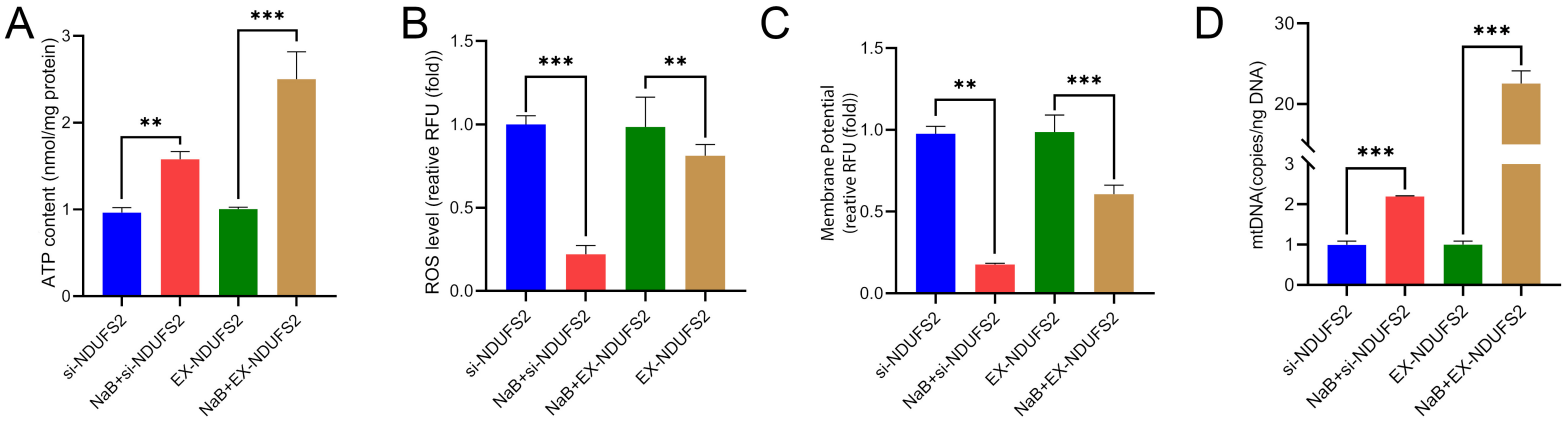
Effect of NaB and *NDUFS2* gene co-treatment on mitochondrial function in BSMCs. **(A)** ATP level. **(B)** ROS level. **(C)** Membrane potential. **(D)** Mitochondrial copy number level. Significance levels are indicated as follows: “ns” for *P* > 0.05, “^**^” for *P* < 0.05, “^***^” for *P* < 0.01.

Co-treatment of NaB with *NDUFS2* gene resulted in a highly significant increase in ATP levels (*P* < 0.05; Figure 6A) and ROS levels (*P* < 0.01; Figure 6B). NaB+si-NDUFS2 significantly reduced the membrane potential (*P* < 0.01), and the membrane potential was reduced in the NaB+EX-NDUFS2 group (*P* < 0.001), suggesting that NaB inhibits the level of the membrane potential (Figure 6C). Co-treatment of NaB with *NDUFS2* elevated mtDNA copy number (*P* < 0.001), suggesting that NaB promotes mtDNA copy number (Figure 6D).

### 3.7 Differential gene screening and functional analysis

**Figure 7:**
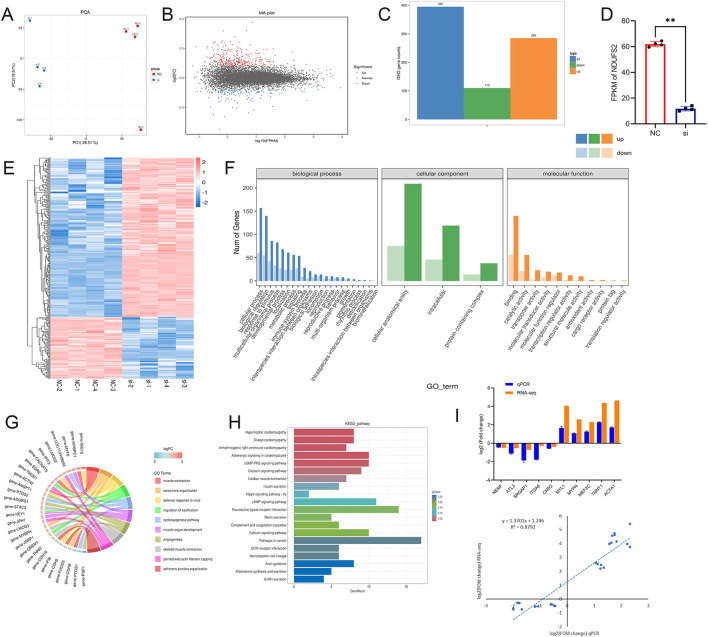
The results of transcriptome sequencing. **(A)** PCA plot. **(B)** M-vs.-A plot of DEGs. Each point in the differentially expressed volcano plot represents a gene; the vertical coordinate represents the logarithmic value of the multiplicity of difference in expression of a particular gene in the two samples; the horizontal coordinate represents the log10 (FPKM) value of DEGs. **(C)** Histogram of differential gene statistics. Horizontal coordinates represent different sets of differential genes, blue is all DEGs, orange is up-regulated genes, green is down-regulated genes, and vertical coordinates represent the number of DEGs. **(D)** RNA-seq results for DUFS2 mRNA levels in the two groups. **(E)** Clustering diagram of DEGs. Note: horizontal coordinates represent the name of the sample and the clustering result of the sample, vertical coordinates represent the DEGs and the clustering result of the genes. Different columns in the graph represent different samples, and different rows represent different genes. The colors represent the mRNA level log10 of the genes in the samples. **(F)** Statistical map of GO annotation classification of DEGs. Horizontal coordinates are GO classifications, vertical coordinates are numbers of genes, and different colors represent different primary classifications to which they belong. **(G)** GO enrichment chord plot of DEGs. Note: the left side of the outer circle represents differential genes, the color of the gene color block represents the magnitude of differential folds, red represents up-regulated genes, blue represents down-regulated genes; the right side of the outer circle is the GO term, different colors represent different term; the connecting lines between genes and GO term represent the enrichment relationship between them. **(H)** Histogram of KEGG enrichment of DEGs. Note: horizontal coordinates are gene number: the number of genes of interest annotated in the entry, and vertical coordinates are each pathway entry. Colors of the bars represent the q_value of the hypergeometric test. **(I)** qPCR to verify differentially expressed genes. The mRNA levels of DEGS were validated by qPCR (top), comparison between RNA-seq and qPCR by correlational analyses (bottom). Significance levels are indicated as follows: “^**^” for *P* < 0.05.

RNA-seq was performed on BSMCs with disrupted *NDUFS2* genes. Principal component analysis (PCA) revealed a clear distinction in sample separation across the various experimental groups, indicating significant disparities in gene expression between these groups (Figure 7A). The M vs. A plot (Figure 7B) demonstrates the DEGs between the SI group and the NC group. A total of 395 DEGs were identified, comprising 285 upregulated genes and 110 downregulated genes (Figure 7C). Notably, that the mRNA level of *NDUFS2* was significantly downregulated in the group treated with siRNAs (*P* < 0.01), consistent with the experimental results (Figure 7D). Hierarchical clustering analysis of differentially expressed genes between the two groups yielded a heatmap that revealed identical expression patterns within each gene group, yet markedly divergent expression patterns between the groups (Figure 7E).

GO enrichment analysis indicated that DEGs were enriched in terms related to metabolic process and protein-containing complexes term (Figure 7F). Additionally, differentially expressed genes were found to be enriched in terms associated with muscle development, including skeletal muscle contraction, muscle contraction, and muscle organ development (Figure 7G). The present study also identified that the genes were enriched in muscle-related KEGG signaling pathways, including the cAMP signaling pathway, cGMP-PKG pathway, and calcium signaling pathway (Figure 7H). Collectively, these results indicate that *NDUFS2* interacts with myogenic-related differentially expressed genes and KEGG signaling pathways. By randomly selecting DEGs from RNA-seq and validating them via qPCR (Figure 7I), the expression trends of these genes were consistent with the RNA-seq results, confirming the reliability of the sequencing data.

### 3.8 *NDUFS2* interacts with NDUFS1

**Figure 8:**
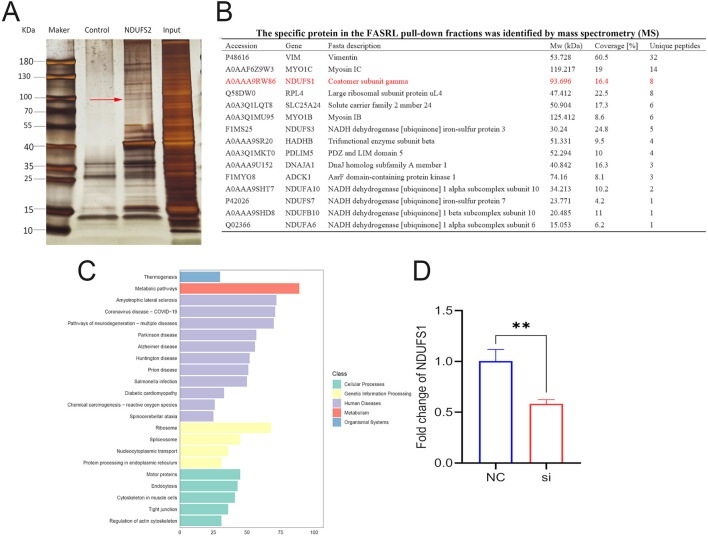
*NDUFS2* binds to *NDUFS1* and promotes its expression. **(A)** Silver staining of RNA pull-down fractions. The arrow indicates the specific protein band from the *NDUFS2* pull-down fractions. **(B)** The specific protein in the FASRL pull-down fractions was identified by mass spectrometry (MS), and the protein marked in red is *NDUFS1*. **(C)** KEGG annotation. The horizontal axis represents the number of proteins identified in this entry, while the vertical axis denotes KEGG pathways. The figure displays only the top 22 KEGG pathways. **(D)** Interference with *NDUFS2* inhibits the *NDUFS1* gene mRNA level. Significance levels are indicated as follows: “^**^” for *P* < 0.05.

To further investigate proteins that directly interact with *NDUFS2*, we employed RNA pull-down assays in conjunction with mass spectrometry (MS) to identify RNA-binding proteins (RBPs). Through DNA amplification and recovery (Supplementary Figure S3A), *in vitro* transcription (Supplementary Figure S3B), and biotin labeling (Supplementary Figure S3C), we identified a total of 994 proteins interacting with *NDUFS2* (Supplementary Figure S3D). Compared to the control, silver staining revealed a distinct band of approximately 100 kDa when RNA was pulled down using the sense strand of *NDUFS2* (Figure 8A). Subsequent MS analysis confirmed the presence of NDUFS1 in the protein fraction derived from the *NDUFS2* sample, which aligned with the silver staining results (Figure 8B). GO and KEGG annotations identified binding proteins associated with metabolic processes and muscular activity (Supplementary Figures S3E–G, Figure 8C). Interference with the *NDUFS2* gene significantly suppressed the mRNA levels of the *NDUFS1* gene (*P* < 0.01; Figure 8D).

## 4 Discussion

Skeletal muscle consists of two distinct fiber types: type I and type II. Type I fibers are classified as “slow-twitch fibers”, a designation derived from the rate of ATP hydrolysis (9, 26). Mitochondrial function encompasses the regulation of ROS production, membrane potential levels, and cellular calcium homeostasis. It also serves as a regulator of fiber type in skeletal muscle tissue (27). The higher the metabolic activity of mitochondria, the greater the likelihood of ROS production (28). However, the role of the mitochondria-associated gene *NDUFS2* in muscle fiber type and mitochondrial function in BSMCs remains unclear.

In this study, mitochondrial indicators were examined, revealing that *NDUFS2* gene promotes the generation of ATP and mtDNA, while simultaneously suppressing levels of ROS and membrane potential in BSMCs (29–32). The knockdown of *NDUFS2* gene significantly reduced cell growth, glycolytic capacity, ATP, and cell membrane integrity in the culture medium, while significantly markedly increasing ROS production, apoptosis, and necrosis (33, 34). *NDUFS2* is located in the inner mitochondrial membrane and plays a pivotal role in maintaining cellular energy metabolism homeostasis, preventing oxidative stress, and ensuring cell survival. Its deletion has been associated with pathological effects (35). Dunham-Snary et al. (21) conducted a knockout experiment in mice, revealing that neural stem cell proliferation and ATP synthesis were significantly impaired. Gerber et al. (36) interfered with *NDUFS2* in pancreatic cancer cell lines, resulting in mitochondrial damage and impaired mitochondrial function. Archer et al. (20) demonstrated that *NDUFS2* contributes to ROS generation in pulmonary artery smooth muscle. The present study shows that *NDUFS2* gene exerts significant regulatory effects on BSMCs, as evidenced by the promotion of cell proliferation and substantial inhibition of their differentiation. Furthermore, the gene exhibited a promotive effect on slow type fibers while showing an inhibitory effect on fast type fibers. Thus, it can therefore be concluded that *NDUFS2* gene has the potential to regulate mitochondrial function and muscle development. These findings confirm that changes in *NDUFS2* expression significantly influence mitochondrial biogenesis, energy metabolism, and myofiber type transition in BSMCs.

**Figure 9:**
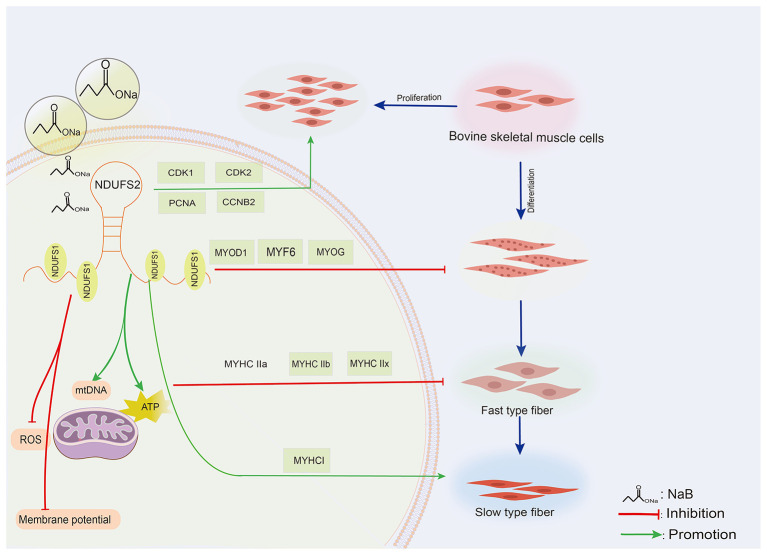
A model describing NaB with *NDUFS2* gene to regulate BSMCs muscle fiber type transformation and mitochondrial biosynthesis.

Mitochondrial function known to be is tightly regulated in relation to myofibrillar type conversion. In the context of animal husbandry, NaB, a common feed additive, holds significant potential for exploring its synergistic effects with endogenous genes. Consequently, we conducted co-treatment with *NDUFS2* and NaB, aiming to investigate the existence of synergistic effects. In a previous study conducted by our research group, we observed that the addition of 1 mm NaB and the induction of cell differentiation for 12 h resulted in the following phenomenon: the intracellular ROS level exhibited an increasing trend, while concurrently, the cellular ATP content and the number of mitochondria increased, although the MMP decreased (14). Our study demonstrates that NaB co-treatment with *NDUFS2* gene promotes mitochondrial ATP production and mtDNA copy number, while simultaneously suppressing ROS levels and membrane potential (Figure 9). The results indicate that co-treatment exhibits significantly stronger effects than the individual functional effects of *NDUFS2* gene. This is primarily manifested in a more pronounced promotion of ATP production and mtDNA copy number, as well as a stronger suppression of ROS levels and membrane potential. This suggests that they may act through distinct pathways and exist within overlapping or complementary regulatory networks, providing new perspectives for identifying novel nutritional strategies to enhance beef quality traits.

As core subunits of mitochondrial complex I (NADH: ubiquinone oxidoreductase), *NDUFS2* and NDUFS1 are structurally interlinked and functionally synergistic, jointly maintaining the complex's stability, assembly integrity, and catalytic activity (37, 38). Evidence suggests a positive feedback regulatory relationship between these subunits, where in the expression level of one can directly influence the protein stability of the other (19). Notably, post-translational modifications of NDUFS1, such as glutathionylation, are known to induce conformational changes in complex I, leading to increased reactive oxygen species (ROS) production under specific conditions, including reverse electron transport (RET). This regulatory modification may also itself be influenced by the local microenvironment mediated by *NDUFS2* (39). Our study observed that significant changes in *NDUFS2* expression affect mitochondrial biogenesis and myofiber type transition, a phenotype likely resulting from its systemic regulation of overall complex I function. Therefore, targeting the *NDUFS2*-NDUFS1 axis could represent a novel strategic avenue for modulating muscle metabolic phenotype and development.

## 5 Conclusion

*NDUFS2* gene, which interacts with the NDUFS1 protein, promotes the proliferation and inhibits differentiation of BSMCs. It enhances the conversion of fast-to-slow type fiber. Additionally, it promotes mitochondrial ATP synthesis and increases the mtDNA copy number, while reducing cellular cellular ROS level and membrane potential. Notably, NaB resulted in more pronounced effects of *NDUFS2* on muscle fiber type transformation and mitochondrial function in BSMCs. Our results elucidate the potential regulatory role of the butyrate-mediated *NDUFS2* in the transformation of muscle fiber types and mitochondrial generation in BSMC.

## Author's note

This study investigated the effects of *NDUFS2* on BSMC proliferation, differentiation, fiber type conversion, and energy metabolism using gene manipulation techniques. Additionally, BSMCs were treated with sodium butyrate (NaB) to assess its impact on NDUFS2 functionality, and protein interactions were examined.

## Data Availability

The datasets presented in this study can be found at: NCBI Sequence Read Archive (SRA), Accession Number (PRJNA1433172): https://www.ncbi.nlm.nih.gov/sra/PRJNA1433172.
