# Supplementary material for: Sodium butyrate promotes the function of NDUFS2 in bovine skeletal muscle fiber type transformation and mitochondrial biosynthesis
Source: Front Vet Sci. 2026 Mar 17;13:1747515. doi: 10.3389/fvets.2026.1747515 (PMC13037377; doi:10.3389/fvets.2026.1747515)
Supplement: Supplementary file 1 [file Data_Sheet_1.docx]

Supplementary Material


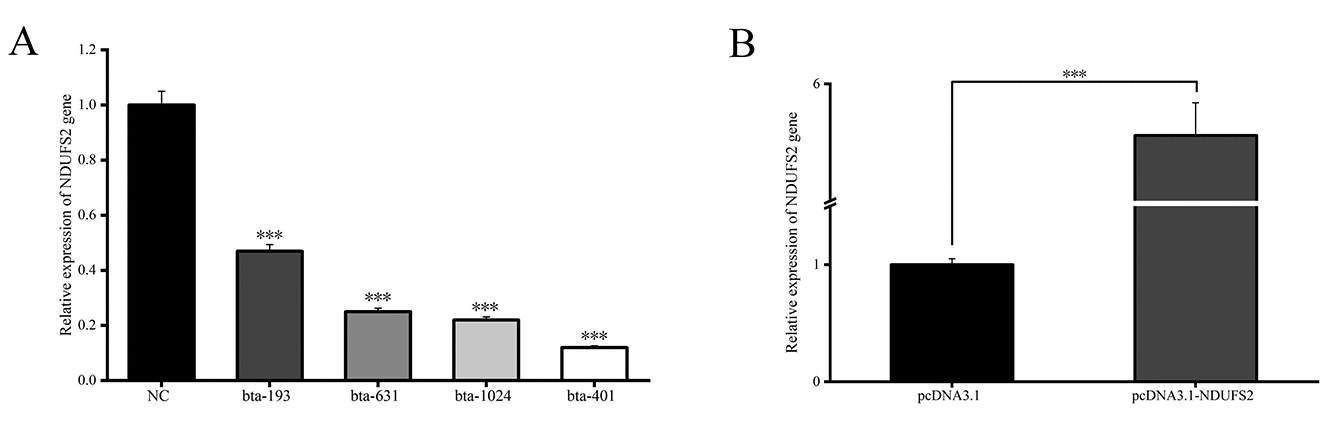


**Supplementary Figure 1.** Validation of NDUFS2 interference and overexpression efficiencies. (A), NDUFS2 gene interference fragment efficiency screening, bta-401 had the highest interference efficiency (*P*<0.01) among the four siRNAs (bta-193, bta-401, bta-631, and bta-1024), which were used as the follow-up experiments. (B), NDUFS2 overexpression efficiencies were highly significantly increased (*P*<0.01).


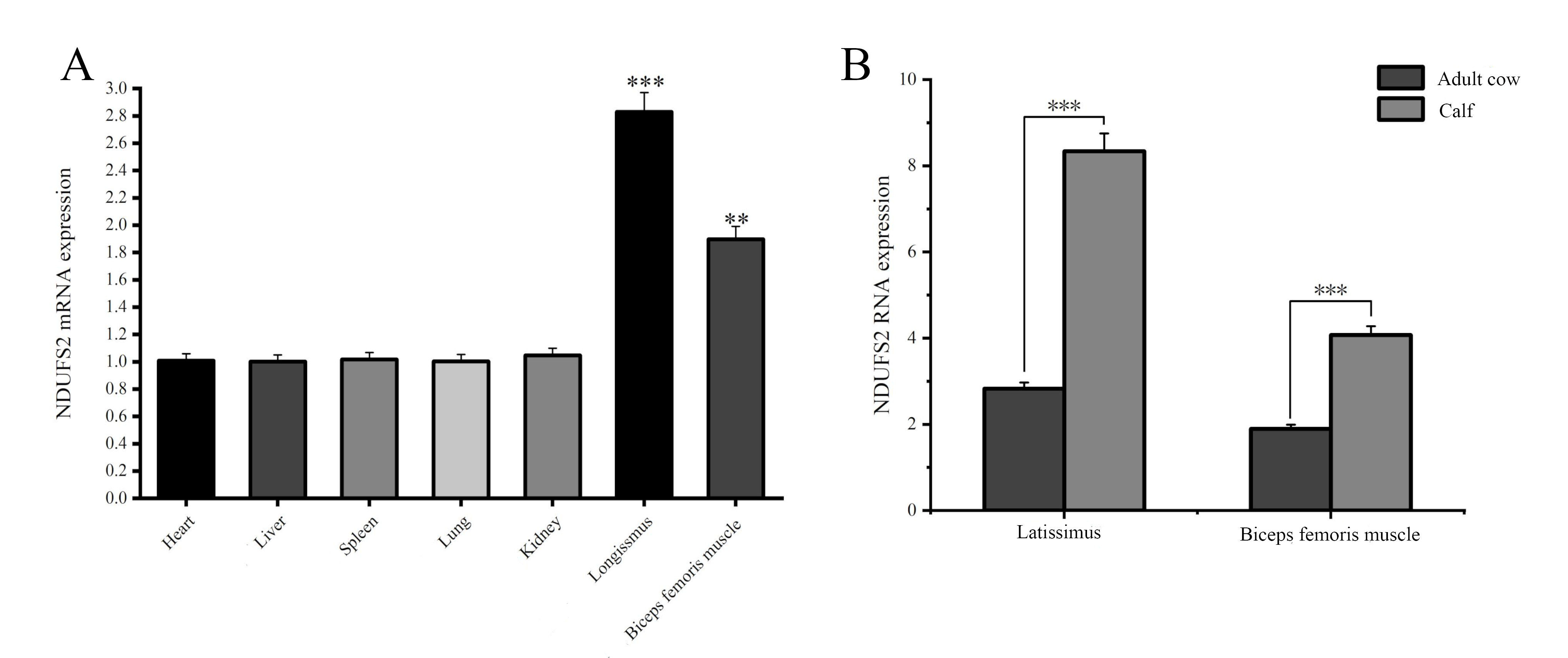


**Supplementary Figure 2.** Tissue expression profile of bovine NDUFS2 gene. (A), mRNA expression levels of NDUFS2 gene in different tissues. (B), difference in mRNA expression of NDUFS2 gene between calf and adult cattle. Note:“**”*P*<0.05 is a significant difference,“***”*P*<0.01 is a highly significant difference.


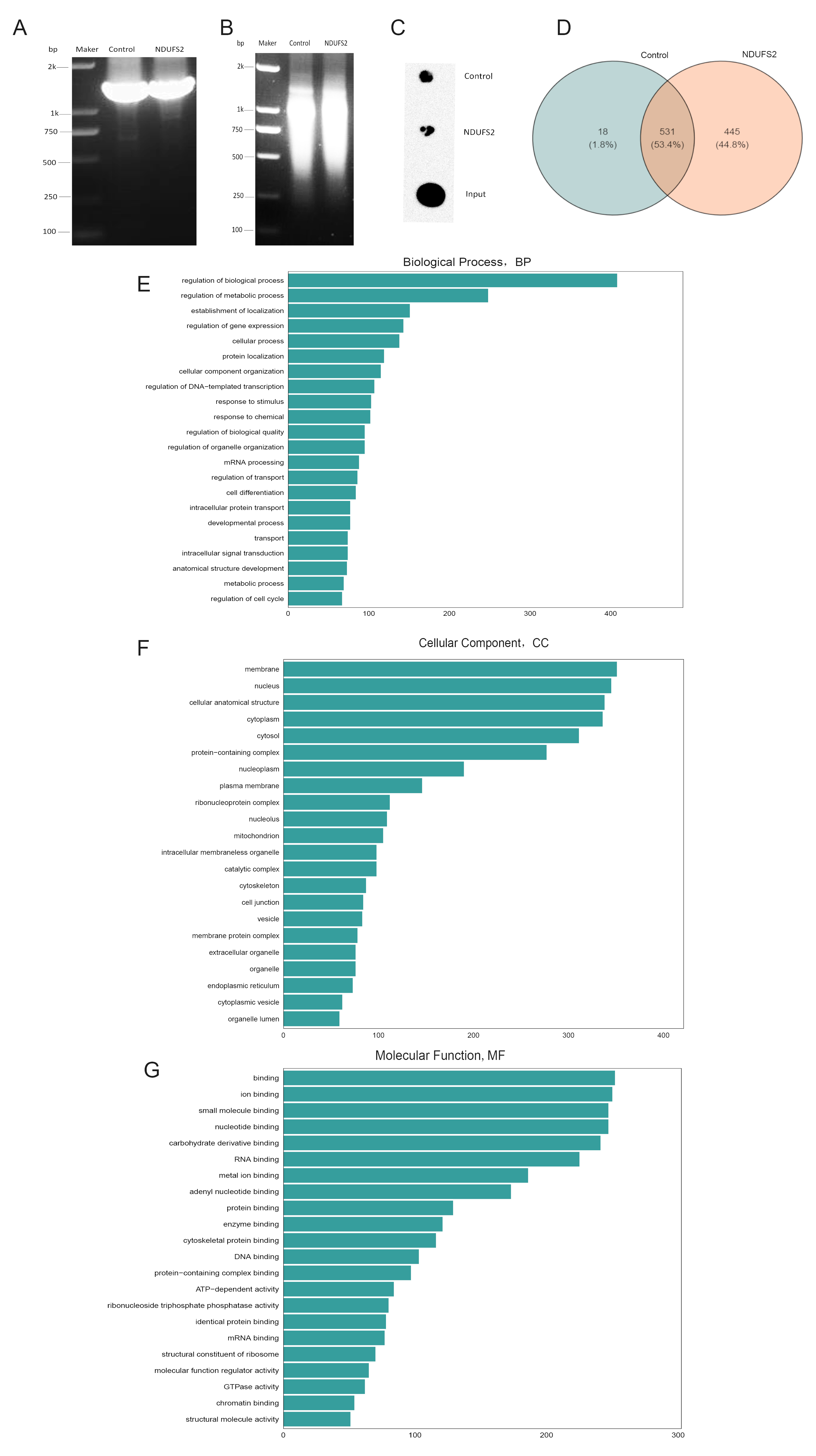


**Supplementary Figure 3.** A total of 994 proteins interacting with NDUFS2 were identified through mass spectrometry (MS). (B), NDUFS2 probe amplification results. The NDUFS2 probe target size is 1392 bp, with the target band successfully amplified. (C), Spot hybridisation. Biotin-labelled RNA is suitable. Biotin-labelled RNA is qualified. (D), Venn diagram. The protein intersections between different samples are shown in the figure. (E-F), GO annotation results. Each row represents a GO term, with the x-axis indicating the number of proteins identified within that term. Only the top 24 GO terms are displayed in the graph.

| **Supplementa**ry **Table S1**. NDUFS2 gene si-RNA sequence design | | |
| --- | --- | --- |
|  | Sense Strand（5'-3'） | Antisense Strand（5'-3'） |
| btaNDUFS2-193 | GGAAACCUCCUCCUUGGAATT | UUCCAAGGAGGAGGUUUCCTT |
| btaNDUFS2-631 | GGGAGAAGAUGUUCGAGUUTT | AACUCGAACAUCUUCUCCCTT |
| btaNDUFS2-1024 | GCCAGUCCAUUCGAAUCAUTT | AUGAUUCGAAUGGACUGGCTT |
| btaNDUFS2-401 | GCAGGCCCUUCCAUACUUUTT | AAAGUAUGGAAGGGCCUGCTT |

| **Supplementary Table S2.** Gene primer information | | | | |
| --- | --- | --- | --- | --- |
| Gene | Accession number | Forward primer sequence（5’-3’） | Reverse primer sequence（5’-3’） | Product length（bp） |
| CDK1 | NM_174016.2 | ACCAAGTTCACCGCCATCAA | TGCACGAACGACACACTCTT | 193 |
| CDK2 | NM_001014934.1 | TCTTTGCTGAGATGGTGACCC | TAACTCCTGGCCAAACCACC | 257 |
| CCNB2 | NM_174264.3 | AAGCTGGCGCTTGGAAGTTA | GTATGGTAGACCCCGGCTTT | 160 |
| PCNA | NM_001034494.1 | GAACCTCACCAGCATGTCCA | ACGTGTCCGCGTTATCTTCA | 86 |
| MYOD1 | [NM_001040478.2](https://www.ncbi.nlm.nih.gov/nuccore/NM_001040478.2) | AACCCCAACCCGATTTACC | CACAACAGTTCCTTCGCCTCT | 196 |
| MYF6 | [NM_181811.2](https://www.ncbi.nlm.nih.gov/nuccore/NM_181811.2) | GTGATAACTGCCAAGGAAGGAG | CGAGGAAATGCTGTCCACGA | 93 |
| MYOG | NM_001111325.1 | GGCGTGTAAGGTGTGTAAG | CTTCTTGAGTCTGCGCTTCT | 85 |
| MyHCⅠ | NM_174727 | TCCCTGATCCACTACGC | TGCCTTTGCCCTTCTC | 178 |
| MyHCⅠⅠa | NM_001166227 | TGGAGCGGATGAAGAAGAACA | GCTTCTGCTCACTCTCTACCTCTC | 159 |
| MyHCIIb | XM_002695806 | AGTGCTATCCCAGAGGGTCAGT | AGCTTTTCATCTCGCATCTCCT | 161 |
| MyHCⅠⅠx | NM_174117 | AGAAGCTGTGAACGCCAAAT | TGTGCTAAGGGAGCGAGACT | 226 |
| GAPDH | NM_001034034.2 | TCGGAGTGAACGGATTCGGC | ATGGCGACGATGTCCACTTT | 82 |

| Supplementary Table S3. Antibody information | | | | |
| --- | --- | --- | --- | --- |
| Name of antibody | Origin | Dilution rate | Cat No. | Company |
| MYF6 Antibody | Rabbit | 1:1000 | A15291 | Abclonal |
| MYOG Antibody | Rabbit | 1:1000 | A17427 | Abclonal |
| MYOD1 Antibody | Rabbit | 1:1000 | CY6736 | Abways |
| MYH4 Antibody | Mouse | 1:1000 | MABT847 | Sigma |
| MYH7 Antibody | Mouse | 1:1000 | SAB2106550 | Sigma |
| Goat Anti-Rabbit IgG (H+L) HRP | Rabbit | 1:2000 | AB0101 | Abways |
| β-actin Antibody | Rabbit | 1:5000 | AB0035 | Abways |
